# The Effects of Quinacrine, Proglumide, and Pentoxifylline on Seizure Activity, Cognitive Deficit, and Oxidative Stress in Rat Lithium-Pilocarpine Model of Status Epilepticus

**DOI:** 10.1155/2014/630509

**Published:** 2014-11-16

**Authors:** Mohammad Ahmad, Gasem M. Abu-Taweel, Ahmad E. Aboshaiqah, Jamaan S. Ajarem

**Affiliations:** ^1^Department of Medical Surgical Nursing, College of Nursing, King Saud University, P.O. Box 642, Riyadh 11421, Saudi Arabia; ^2^Department of Basic Sciences, College of Education, Dammam University, P.O. Box 2375, Dammam 31451, Saudi Arabia; ^3^Department of Nursing Administration and Education, College of Nursing, King Saud University, P.O. Box 642, Riyadh 11421, Saudi Arabia; ^4^Department of Zoology, College of Science, King Saud University, P.O. Box 2455, Riyadh 11451, Saudi Arabia

## Abstract

The present data indicate that status epilepticus (SE) induced in adult rats is associated with cognitive dysfunctions and cerebral oxidative stress (OS). This has been demonstrated using lithium-pilocarpine (Li-Pc) model of SE. OS occurring in hippocampus and striatum of mature brain following SE is apparently due to both the increased free radicals production and the limited antioxidant defense. Pronounced alterations were noticed in the enzymatic, glutathione-S transferase (GST), catalase (CAT), and superoxide dismutase (SOD), as well as in the nonenzymatic; thiobarbituric acid (TBARS) and reduced glutathione (GST), indices of OS in the hippocampus and striatum of SE induced animals. Quinacrine (Qcn), proglumide (Pgm), and pentoxifylline (Ptx) administered to animals before inducing SE, were significantly effective in ameliorating the seizure activities, cognitive dysfunctions, and cerebral OS. The findings suggest that all the drugs were effective in the order of Ptx < Pgm < Qcn indicating that these drugs are potentially antiepileptic as well as antioxidant; however, further studies are needed to establish this fact. It can be assumed that these antiepileptic substances with antioxidant properties combined with conventional therapies might provide a beneficial effect in treatment of epilepsy through ameliorating the cerebral OS.

## 1. Introduction

Status epilepticus (SE) is an emergency neurological condition where recurrent generalized seizures last for more than 30 minutes and if not controlled neuronal injury occurs [1, 2]. Besides neurobehavioral deficits, SE is preferentially associated with a wide range of neurochemical imbalance in some areas of the brain [3–6]. Such neuronal hyperactivity and/or excitotoxicity have been associated with excessive generation of free radicals [7, 8], particularly in the brain, which contains large quantities of oxidizable lipids and metals, and moreover, the brain has fewer mechanisms of antioxidation than other tissues [9]. Oxidative stress, which is generally defined as the overproduction of free radicals, can dramatically alter neuronal function and has been linked to the pathogenesis of epilepsy [8, 10–12]. There exist various endogenous antioxidant defense mechanisms, both enzymatic and nonenzymatic, which within certain limits can counteract increased ROS production.

Several neurochemical studies in animal models have revealed that oxidative stress-related seizures produce changes in antioxidant enzymatic activity and receptor binding [8, 13, 14]. The pathological process and underlying mechanisms involved in the oxidative stress during SE are still far from clear. There is ample evidence to suggest that brain tissues are highly vulnerable to the oxidative stress [15]. Reactive oxygen species (ROS) can dramatically affect the structure and function of neurons and recently Freitas et al. [15] suggested an evidence based role of ROS in the pathophysiology of SE. Importantly, epilepsy induced by pilocarpine in rodent models can provide information regarding oxidative stress-related epileptic activity [3, 4, 16–18]. Besides epileptic seizure activities, oxidative stress has also been related with cognitive impairment [19–22]. Furthermore, in such animal models, pharmacological approaches designed to reduce the oxidative stress afford significant neuroprotection, attesting to the critical role that oxidative stress plays in a diverse array of neurological diseases and disorders. Inhibition of the neurochemical pathways responsible for neuronal excitability and behavioral changes may lead to the development of new therapeutic agents for the treatment of SE.

The anticonvulsant effect of several agents having antioxidant property such as curcumin, vineatrol, transresveratrol, melatonin, adenosine, alpha lipoic acid, pentoxifylline, buspirone, aloe vera, and metformin has been demonstrated in various studies [2, 6, 23–28].

Proglumide (Pgm) is a known cholecystokinin (CCK) antagonist [29] and changes CCK level and receptor population of CCK has been associated with SE [30]. Quinacrine (Qcn) is a potent phospholipase A2 (PLA2) inhibitor [31] and has been shown to exert neuroprotective activity in ischemic brain [32]. PLA2 are a family of ubiquitous enzymes that degrade membrane phospholipids and produce a variety of lipid mediators to regulate neuronal functions [32]. PLA2 has also been shown to have a direct role in regulation of neurotransmitters and electrolytes in normal and reduced OS-mediated cellular toxicity. Pentoxifylline (Ptx) has also been shown to have neuroprotective effects against a host of neurobehavioral disorders including ischemic brain injury, neurotrauma, dementia, stroke, improved cognitive effect following hippocampal lesions, and ameliorated effects on lithium-pilocarpine induced SE in young rats [6, 33–35].

The pilocarpine- (Pc-) induced seizure in rodents is generally considered as one of the most suitable experimental models that has been frequently used to study the pathophysiology and management strategies of SE [3, 8, 36]. Due to high mortality of animals in pilocarpine (alone) model [37], a combination of lithium chloride and pilocarpine (Li-Pc) model is often preferred. Lithium (Li) potentiates the epileptogenic action of Pc but at the same time significantly reduces the mortality of animals [38, 39]. The Li-Pc model of SE reproduces most clinical, temporal, and neuropathological features of SE [3, 6, 40, 41].

The present study was designed to study the comparative effects of Pgm, Qcn, and Ptx on epileptic seizure activities, cognitive tests, and oxidative stress in the brain regions of rats in which SE was induced by lithium-pilocarpine. Our prime objective was to assess if the oxidative stress observations in the SE animals can be of any help in identifying effective antiepileptic drugs.

## 2. Materials and Methods

### 2.1. Experimental Animals

The animals used in the present study were adult male Sprague Dawley rats (weighing 200–250 g, 2 months old) and were housed under controlled conditions with 12 hours light-dark diurnal cycle at 22 ± 1°C, with humidity at 50–60% and with free access to food and water except during experimental handlings. All study protocols and animal handling procedures were in accordance with and approved by the Research and Ethics Committee of King Saud University, Riyadh, Saudi Arabia. The prior permission was obtained from this committee for executing the experiments.

### 2.2. Induction of SE

Animals were randomly assigned into thirteen groups. The animals in groups 1, 2, and 3 served as controls and received saline, Li (3 mEq/mL/kg,* i.p.*), and Pc alone (20 mg/mL/kg,* s.c.*), respectively. SE was induced in groups 4 to 13 by administering an aqueous (saline) solution of Li (BDH Laboratory Supplies, Poole, England, in a dose as in control), followed by (20 h later) Pc (Sigma Chemical Co., St. Louis, MO, USA, in the dose as used for control). Group 4 served as the experimental control of SE group and groups 5 to 13 served as the drug test groups. Qcn, Pgm, and Ptx (Sigma, USA) were dissolved in saline and were administered at doses of 5, 15, and 30 mg/mL/kg* i.p.*; 250, 500, and 750 mg/mL/kg* i.p.*; and 20, 40, and 60 mg/mL/kg* i.p.* (one hour before Pc injection), to groups 5 to 13, respectively. Groups receiving Qcn, Pgm, and Ptx alone in the highest dose (30, 750, and 60 mg/kg, resp.) served as the controls for the drugs used in this study. However, the results of these drug alone controls are not included in the results of this study, since these drugs administered alone in the animals did not show any abnormal behavioral activities and resembled the untreated controls. Furthermore, even all investigated parameters in this study for the drug alone groups were almost similar as the naïve control groups. Thus none of the results for the drug alone treated groups are included in the results of this study in order to avoid unnecessary confusion and crowding in the numbers of bars shown in the result figures. After Pc injections, the animals (*n* = 10 per group) were observed for a sequence of behavioral alterations, including peripheral cholinergic signs (PCS), stereotyped movements (STM), clonic movements of forelimbs, head bobbing, tremors, and seizures, which developed progressively within 1-2 h into SE [42]. For comparative observations on the drug effects, all seizure activities were presented as latency to develop seizure and SE. The seizure behavior consists of head bobbing with intermittent forelimb and hind limb clonus, hyperextension of tails, loss of posture, falling back, and myoclonic jerks, whereas SE is a condition where these recurrent generalized seizures last for more than 30 minutes in the animals. Mortality (if any) within 24 h was also recorded.

#### 2.2.1. Morris Water-Maze Test

After induction of SE for seizure activities analyses, the animals (*n* = 6 to 8 per group) were subjected to cognitive test. Animals were allowed to acclimatize to the testing room for 2 h before testing. All tests were performed between 10:00 and 15:00 hours of the lighted phase.

The test has been extensively used to assess cognitive functions in a variety of epilepsy models [43, 44]. The rats were tested for visual-spatial memory using a water-maze [45]. The water-maze consisted of a galvanized white circular water tank (117 cm diameter, 55 cm height) filled with clear tap water (26 ± 1°C) to a depth of 30 cm. A 10 cm diameter, stainless steel, white, escape platform was placed 1 cm below the water level and the water was made opaque by addition of 1 liter of milk, which prevented visualization of the platform. Four points on the rim of the tank were designated north (N), south (S), east (E), and west (W), thus dividing the pool into four quadrants (NW, NE, SE, and SW).

On the first day, each rat was allowed to swim freely in the pool for 60 sec without the platform present in the pool. This free swim enabled the rat to become habituated to the training environment. On days 2–5, rats were trained for 24 trials (six trials a day, with an intertrial interval of 30 sec) to locate and escape onto the submerged platform. At the start of each trial, the rat was held facing the perimeter of the water tank and dropped into the pool to ensure immersion. The latency from immersion into the pool to escape onto the hidden platform (maximum trial duration 120 sec) was recorded. On mounting the platform, each rat was given a 30 sec intertrial interval for rest and for learning and memorizing the spatial cues to reach the platform for escape. The testing procedures used during the four days of locating the hidden platform provide a measure of hippocampal-dependent spatial reference memory [46].

### 2.3. Biochemical Studies

Based on our pilot studies and literature survey [5, 47, 48], the biochemical studies were undertaken 1 h after Li-Pc treatment and immediately after killing the rest of the animals by decapitation, their brains were dissected on ice, and cerebral areas (hippocampus and striatum) were removed and frozen in liquid nitrogen and stored at −70°C for determination of some nonenzymatic and enzymatic oxidative stress indices.

### 2.4. Determination of Nonenzymatic OS Indices

#### 2.4.1. Lipid Peroxides

Lipid peroxides (LP) in striatum and hippocampus were determined spectrophotometrically as thiobarbituric acid-reactive substances (TBARS) according to the method of Ohkawa et al. [49]. Tissue lipid peroxide levels were quantified using extinction coefficient of 1.56 × 10^5^ m^−1 ^cm^−1^ and expressed as nanomoles of TBARS formed per g tissue weight. The results are expressed as nmol/g wet weight.

#### 2.4.2. Glutathione

Reduced glutathione (GSH) level in striatum and hippocampus was measured enzymatically in the brain tissues by a slightly modified method [50]. The slope of the change in absorbance was used to quantitate total GSH by comparing the slope of the samples with a standard curve prepared with pure glutathione (Sigma). The specific activity is expressed into umol/g tissue weight.

### 2.5. Determination of Enzymatic OS Indices

#### 2.5.1. Glutathione-S-Transferase

Glutathione S-transferase (GST) was estimated by the method of Habig et al. [51] by using 1-chloro-2,4-dinitrochlorobenzene (CDNB) as substrate at 340 nm. The GST activity is expressed as U/g tissue weight.

#### 2.5.2. Catalase

Catalase (CAT) activity was measured by the method of Aebi [52], by tracking the decomposition of hydrogen peroxide by measuring decrease in extinction of H_2_O_2_ at 240 nm. The activity of CAT is expressed as rate constant of first order reaction K per gram tissue weight.

#### 2.5.3. Superoxide Dismutase

Superoxide dismutase (SOD) activity was estimated by the method of Misra and Fridovich [53]. Activity is expressed as the amount of enzyme that inhibits the oxidation of epinephrine by 50% which is equal to U per gram tissue weight.

### 2.6. Statistical Analysis

The data were analyzed by Bartlett's test for equal variance and by Gaussian-shaped distribution for normality using the Kolmogorov-Smirnov goodness-of-fit test. As the data passed the normality test (*P* > 0.10), group means were compared with one-way ANOVA with post hoc testing using Tukey-Kramer Multiple Comparisons Test or Student-Newman-Keuls Multiple Comparisons Tests. Differences in seizure and SE incidences and mortality were tested by Student-Newman-Keuls Multiple Comparisons Tests. All results were expressed as means ± SEM and the significance was defined as *P* < 0.05 for all tests.

## 3. Results

### 3.1. Features of Li-Pc Induced SE

Within 5 min after injection of Pc, all animals started developing a gradual and significant change in behavior including PCS (miosis, piloerection, diarrohea, mild tremors, scratching, and salivation) and STM (sniffing, paw licking, and rearing) followed by seizures in 100% of the animals with a mean latency of 9.62 ± 1.2 min to develop seizure (Table 1). The convulsive episode consisted of head bobbing with intermittent forelimb and hind limb clonus, hyperextension of tails, loss of posture, falling back, and myoclonic jerks building up to SE in 100% of animals. The mean latency to onset of SE was 23.86 ± 1.54 min (Table 1), and on average, the SE lasted for more than one hour. A total of 10% mortality were observed over a period of 24 h following Pc injections (Table 1).

### 3.2. Effect of Drugs Pretreatment on Li-Pc Induced SE

Pgm, Qcn, and Ptx dose-dependently and significantly increased the latencies to seizure and SE and decreased the percentages of seizures and SE (Table 1) and also reduced the intensity and frequency of seizure, PCS, and STM episodes (not shown in Table 1). The severity of SE was significantly and dose-dependently reduced and latency to attain SE was increased in the drug treated animals. The drugs were effective in the order Ptx > Pgm > Qcn. The highest dose (60 mg/kg) of Ptx completely abolished SE (Table 1). Furthermore, no mortality was observed in the rats pretreated with all the three drugs, as compared to 10% mortality in the Li-Pc treated group (Table 1). The control groups that received Li or the drugs alone did not show any signs of seizure or SE.

### 3.3. Morris Water-Maze Test

**Figure 1:**
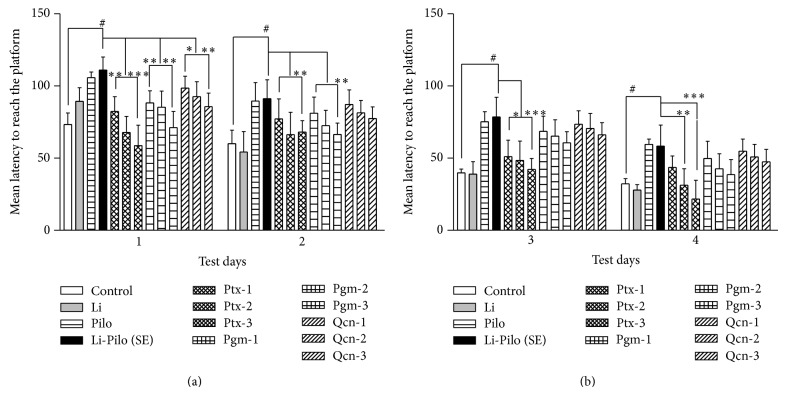
Performance in water-maze of animals that experienced SE along with their controls as well as the SE groups that were pretreated with Ptx, Qcn, and Pgm. Mean latency to reach the hidden platform in seconds ± SEM (*y*-axis) on each testing day (*x*-axis) shows that animals subjected to SE were slower in finding the platform (cognitive effect) than the controls on all four testing days. Treatment with Ptx, Qcn, and Pgm in varying doses was effective in improving the cognitive function in the order Ptx < Pgm < Qcn and dose-dependently. Li = lithium chloride; Pilo = pilocarpine; SE = status epilepticus; doses of drugs in mg/kg body weight: Ptx-1, Ptx-2, and Ptx-3 = 20, 40, and 60; Pgm-1, Pgm2, and Pgm3 = 250, 500, and 750; and Qcn-1, Qcn-2, and Qcn-3 = 5, 15, and 30, respectively. # represents significance as compared to control (*P* < 0.001), whereas ∗, ∗∗ and ∗∗∗ show *P* < 0.01, *P* < 0.05, and *P* < 0.001, respectively, as compared to SE group by one-way ANOVA.

Rats with Li-Pc treatment exhibited longer escape latencies to reach the platform as compared with control group (*P* < 0.01; Figure 1); however, all groups displayed gradual improvement in performance over the 4 days of testing (training) period. The number of successful animals to reach the platform was significantly higher in the drug pretreated groups as compared to Li-Pc (SE) group on all the four testing days but in the order Ptx > Pgm > Qcn (*P* < 0.001; Figure 1).

### 3.4. Biochemical Studies

#### 3.4.1. Nonenzymatic OS Indices

**Figure 2:**
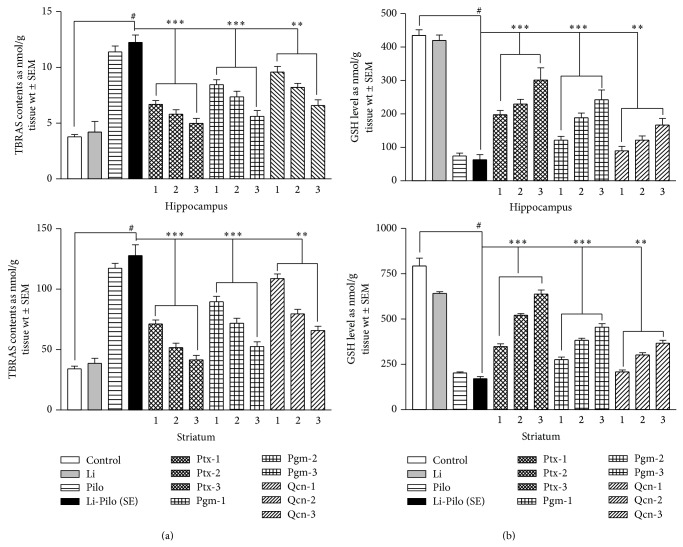
Comparative effect of Ptx, Pgm, and Qcn on the nonenzymatic oxidative stress indices like (a) lipid peroxidation content (TBARS) and (b) total glutathione level (GSH), in hippocampus and striatum of rats after Li-Pc induced SE. The comparative effects are in the order Ptx < Pgm < Qcn and dose-dependent. Abbreviations and statistical significance are the same as in Figure 1.

*TBARS in Hippocampus and Striatum*. The lipid peroxidation level (TBARS) in the hippocampus and striatum was markedly (*P* < 0.001) increased after 1 h of Li-PC (SE) treatment as compared to the control group (Figure 2(a)). Pretreatment with drugs significantly (*P* < 0.001) and dose-dependently attenuated Li-Pc induced increase in TBARS in the hippocampus and striatum in the order Ptx > Pgm > Qcn (Figure 2(a)) as compared to Li-Pc (SE) group.

*GSH in Hippocampus and Striatum*. A highly significant (*P* < 0.001) depletion of hippocampal and striatal GSH was observed in Li-Pc (SE) group (Figure 2(b)). Pretreatment with drugs significantly and dose-dependently attenuated this depletion of GSH in the hippocampus and striatum in the order Ptx > Pgm > Qcn (Figure 2(b)) as compared to Li-Pc (SE) group.

#### 3.4.2. Enzymatic OS Indices

**Figure 3:**
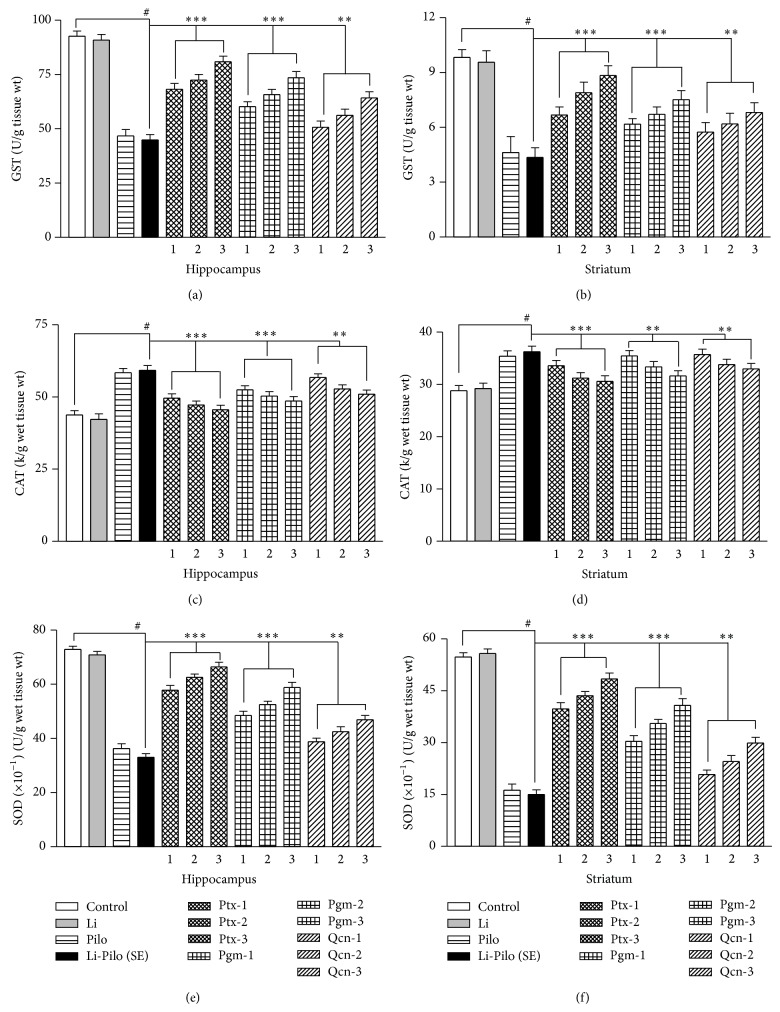
Comparative effect of Ptx, Pgm, and Qcn on the enzymatic oxidative stress indices like glutathione-S-transferase level (GST) in hippocampus (a) and striatum (b); catalase (CAT) activity in hippocampus (c) and striatum (d) and superoxide dismutase (SOD) activity in hippocampus (e) and striatum (f) of rats after Li-Pc induced SE. The comparative and dose-dependent effects are in the order Ptx < Pgm < Qcn. Abbreviations and statistical significance are the same as in Figure 1.

*GST in Hippocampus and Striatum*. A highly significant (*P* < 0.001) depletion of hippocampal (Figure 3(a)) and striatal (Figure 3(b)) GST was also observed in Li-Pc (SE) group. Pretreatment with drugs significantly and dose-dependently attenuated this depletion of GST in the hippocampus and striatum in the order Ptx > Pgm > Qcn (Figures 3(a) and 3(b)) as compared to Li-Pc (SE) group.

*CAT in Hippocampus and Striatum*. The CAT level in the hippocampus (Figure 3(c)) and striatum (Figure 3(e)) was markedly (*P* < 0.001) increased after 1 h of Li-PC (SE) treatment as compared to the control group (Figures 3(c) and 3(d)). Pretreatment with drugs significantly (*P* < 0.001) and dose-dependently attenuated Li-Pc induced increase in CAT in the hippocampus and striatum in the order Ptx > Pgm > Qcn (Figures 3(c) and 3(d)) as compared to Li-Pc (SE) group.

*SOD in Hippocampus and Striatum*. The SOD level in the hippocampus (Figure 3(e)) and striatum (Figure 3(f)) was significantly (*P* < 0.001) decreased after 1 h of Li-PC (SE) treatment as compared to the control group. Pretreatment with drugs significantly (*P* < 0.001) and dose-dependently attenuated Li-Pc induced decrease in SOD in the hippocampus and striatum in the order Ptx > Pgm > Qcn (Figures 3(e) and 3(f)) as compared to Li-Pc (SE) group.

## 4. Discussion

The present findings indicate that Pc administration to rats pretreated with Li initiated cholinergic symptoms including miosis, piloerection, diarrhea, and mild tremors followed by seizures. SE developed between 20 to 30 minutes after Pc administration which consisted of head bobbing, intermittent forelimb and hind limb clonus, and hyperextension of tail and hind limb along with loss of posture. No mortality was found in any of the drug treated groups as compared to 10% mortality in the Li-Pc (SE) group. The present Morris water-maze results showed that rats with SE took longer time to reach escape platform, spent lesser time in the target quadrant, or completely failed to reach the platform clearly suggesting impaired visual-spatial memory and cognitive deficit. Cognitive dysfunctions were also evident from the fact that the animals swam along with the wall of the test tank and rarely tried to find the escape platform in the target quadrant. Impairment of learning and memory in rats with SE has been reported by several investigators [6, 54, 55]. The specific cause of motor and cognitive deterioration following SE is far from clear. However, according to recent reports neurochemical imbalance and alteration of neuronal structure following SE might be responsible for neurobehavioral changes [39, 47]. Cha et al. [56] reported that hippocampus receives dense cholinergic projections and overactivation of these afferents by Pc may directly produce neuronal hyperexcitation and trigger seizure activity accompanied by alterations in neuronal plasticity within the hippocampal circuitry causing impairment of learning and memory in rats [44, 57, 58].

The results of the present study clearly showed the antiepileptic activity of all three drugs (Pgm, Qcn, and Ptx) against Li-Pc induced seizure, as revealed by highly significant decrease in frequency of epileptic episodes and increase in the latency to SE. The mortality was absent in all the three drug treated groups and the comparative efficacy of the tested drugs was in the order Ptx > Pgm > Qcn (Table 1). Furthermore, our study consistently demonstrated that pharmacological intervention using Pgm, Ptx, and Qcn significantly and dose-dependently attenuated SE induced impaired memory and the comparative efficacy of the tested drugs was in the same order Ptx > Pgm > Qcn throughout.

The present biochemical studies indicated a significant and dose-dependent increase in TBARS and CAT (Figures 2(a) and 2(b)) and decrease in GSH, GST, and SOD levels (Figures 3(a)–3(f)) in the hippocampus and striatum of the rats treated with Li-Pc clearly suggesting a high level of enzymatic (GST, SOD, and CAT) and nonenzymatic (TBARS and GSH) oxidative stress in these brain regions. It is widely accepted that tissue injury is dependent not only on the nature of offending pathogen but also on the quality of host defense system including the type and levels of antioxidants. Oxidative stress is particularly facilitated in brain since the neuronal cells contain large quantities of oxidizable lipids and metals and the antioxidant defense mechanism in brain is relatively weaker as compared to other organs [8]. The drugs used in the present study significantly and dose-dependently attenuated Li-Pc induced OS in hippocampus and striatum but in the order Ptx > Pgm > Qcn (Figures 2 and 3). Earlier, it has been reported [6] that Ptx may exert its pharmacological effects by several mechanisms including inhibition of phosphodiesterase enzymes (PDEs), leading to increases in cyclic adenosine monophosphate (cAMP), blockade of adenosine receptors, translocation of extracellular calcium, an inhibitory effect on inflammatory mechanism, and free radical scavenging activity. The role of cAMP in the etiopathology of epileptic seizures has been widely studied [59, 60]. Besides its definite association with seizure activity, cAMP also plays a key role in biochemical regulation of the cognitive process of memory consolidation [61]. Earlier studies have also revealed that OS-related seizures produce changes in antioxidant enzyme activities [6, 8, 13, 14] and still other studies have related Pc-induced epileptic activity with the disturbance in OS activities [3, 4, 16]. Neuronal hyperexcitability and excessive production of free radicals have been implicated in the pathogenesis of a considerable range of neurological disorders, including epilepsy [62–64]. Lipid peroxidation in a tissue is an index of irreversible biological damage of the cell membrane phospholipid, which in turn leads to inhibition of most of the sulphydryl and some nonsulphydryl enzymes [26, 65]. Lipid peroxidation can be induced by many chemicals like kainic acid, Pc, and tissue injuries, and it has been suggested for a possible mechanism for the neurotoxic effects observed during epileptic activity [5, 26]. The present findings clearly indicated that lipid peroxidation levels (TBARS) in the striatum and hippocampus of rats were increased and the reduced glutathione (GSH) concentration was decreased after seizure activities of SE induced by Li-Pc. In normal conditions, there is a steady state balance between the ROS production (TBARS) and their scavenging by the cellular antioxidant system (GSH). Presence of Pc may be associated with marked alterations of enzymatic (SOD, CAT, and GST) and nonenzymatic components (TBARS and GSH) of antioxidant defense system (AOS). The comparative study of the three drugs (Qcn, Pgm, and Ptx) demonstrates that these drugs act as effective antioxidants and effectively protect against Pc-induced lipid peroxidation and ameliorate the negative effect of Pc on antioxidant status by protecting the effects from oxidative stress and have an ameliorating effect on the AOS. Thus it is likely that this pathomechanism may contribute at least in part to the pathophysiology of the seizure activity. Antioxidant therapies have been of great interest in the treatment of neurodegenerative disorders [66]. Among the three drugs studied herein, Ptx is found to have a 100% survival rate in the SE animals without any mortality and has the highest neuroprotection from SE in the cognitive behavioral and biochemical parameters as observed herein and as reported in an earlier study [6]. Ptx has been reported as a potent free radical scavenger [67] and the neuroprotective effect of Ptx has been attributed to its antioxidant activity by earlier investigators [34, 35]. The present study clearly suggests that Ptx, Qcn, and Pgm are potent free radical scavengers and have antiepileptic as well as antioxidant activity in the order Ptx > Pgm > Qcn. Antioxidants and free radical scavengers have been shown to reduce neuropathology associated with SE [6].

## 5. Conclusion

In conclusion, the present study suggests that all the drugs used in the present study have potential for being antiepileptic as well as antioxidant in an effective order of Ptx < Pgm < Qcn. However, further studies can confirm these effects and also can indicate whether OS plays a definite role in the pathophysiology of installation and/or propagation of epileptic seizures. It is however assumed that Ptx, Pgm, and Qcn combined with conventional therapies might provide a beneficial effect in the treatment of epilepsy through ameliorating the cerebral OS.

## Figures and Tables

**Table 1:** Dose-dependent antiepileptic activity of pentoxifylline (Ptx), proglumide (Pgm), and quinacrine (Qcn) against Li-Pc induced status epilepticus (SE) in adult rats.

Behavioral parameters observed	Control	Ptx (mg/kg)			Pgm (mg/kg)			Qcn (mg/kg)		
		20	40	60	250	500	750	5	15	30
Latency to seizures (min)	9.62 ± 1.20	14.59^ns^ ± 1.42	24.62^a^ ± 2.44	38.15^a^ ± 1.80	12.46^ns^ ± 1.17	22.47^a^ ± 1.59	35.33^a^ ± 1.64	10.21^ns^ ± 1.42	19.38^a^ ± 2.61	31.72^a^ ± 1.53
Seizures (%)	100	85.0	55.0^a^	10.0^a^	85.2	61.3^a^	21.3^a^	87.4	66.5^a^	29.8^a^
Latency to SE (min)	23.86 ± 1.54	34.61^a^ ± 0.86	48.70^a^ ± 0.96	No SE	32.52^a^ ± 1.07	44.61^a^ ± 1.22	56.11^a^ ± 2.63	29.62^a^ ± 1.61	40.33^a^ ± 2.57	58.8^a^ ± 1.91
SE (%)	100	50.0^a^	20.0^a^	0^a^	61.4^a^	36.8^a^	23.5^a^	68.3^a^	39.2^a^	25.4^a^
Mortality (%) within 24 hours	10	0	0	0	0	0	0	0	0	0

Animals were observed for more than 1 h after Li-Pc (lithium-pilocarpine) injections for inducing SE in all groups, and all animals were observed for more than 24 hours for mortality.

^ns^Statistically nonsignificant.

^a^ *P* < 0.001 as compared to control (0 mg/kg) by ANOVA followed by Student-Newman-Keuls Multiple Comparisons Test.
